# Complications after cryosurgery with new miniature cryoprobes in long hollow bones: An animal trial

**DOI:** 10.1186/1471-2482-5-17

**Published:** 2005-08-07

**Authors:** Frank Popken, Peter Meschede, Heike Erberich, Timmo Koy, Marfalda Bosse, Jürgen H Fischer, Peer Eysel

**Affiliations:** 1 Department of Orthopaedic Surgery, University of Cologne, Josef-Stelzmann-Str. 9, 50931 Cologne, Germany; 2 Institute of Pathology, University of Cologne, Josef-Stelzmann-Str. 9, 50931 Cologne, Germany; 3 Institute of Experimental Medicine, University of Cologne, Robert-Koch-Str. 10, 50931 Köln, Germany

## Abstract

**Background:**

In vitro studies show that new miniature cryoprobes are suitable for cryoablation of bone tissue. The aim of this animal trial on 24 sheep was to examine the perioperative complications, particularly the danger of embolism, of cryoablation when using miniature cryoprobes.

**Methods:**

Cryoablations with 2 freeze-thaw cycles each were carried out in the epiphysis of the right tibia and the metaphysis of the left femur. Pulmonary artery pressure (PAP) and central venous pressure (CVP) were measured. Throughout the intra- and perioperative phase, heart rate and oxygen saturation by pulse oxymetry, blood gas and electrolytes were monitored regularly. Postoperative complications were examined up to 24 weeks postoperativ.

**Results:**

As result, no significant increase of PAP, CVP or heart rate were observed. Blood gases were unremarkable, with pO_2 _and pCO_2 _remaining constant throughout the operation. Regarding pH, standard bicarbonate and base excess, only a non-significant shift towards a slight acidosis was seen. There was a mean hemoglobin decrease of 0.5 g/dl. One animal showed postoperative wound infection and wound edge necrosis. No major peri- and postoperative complications associated with cryosurgery of bone were observed, especially regarding clinically relevant pulmonary embolism.

**Conclusion:**

Surgery with new types of miniature cryoprobes appears to be a safe alternative to or a complement to conventional resection of abnormal bone tissue.

## Background

Surgical treatment of bone tumours often requires generous resection of bone, leaving defects which are difficult to span. Freezing tumours with liquid nitrogen was introduced in the late 1960's as an adjuvant treatment to extend the surgical margin of excision for intralesional resection or for curettage by pouring or spraying the nitrogen directly into the bone cavity [1-9]. Animal trials by Gage et al. [10] have shown that devitalised bone matrix can serve as a framework for new periostal and endostal growth, and hence that the former tumour space can be bridged with autologous, healthy bone tissue. However, the freezing procedure is difficult to control, and therefore harbours risks of injury for the patient [11,12] and the surgical team, as well as of gas embolisms caused by evaporation [13] and spread of the liquid nitrogen.

Aside from the open use of liquid nitrogen, closed systems for treating bone tumours have also been used [14], although they never became popular because the cooling power of the cryoprobes then used was low compared to their diameter [15]. Recent technical advances [16] made it possible for us to develop new probes for cryoablation of bone tissue and test these in animal trials [17,18]. The efficiency of these procedure and the extent of tissue distruction is well documented in a former study with a comparable setup [19].

Various complications have been reported, ranging from soft tissue wound infection and fractures [20] to bone marrow and fat embolism caused by the spread of the ice front due to an increase in intramedullar pressure [21]. These miniature cryoprobes with a minimised diameter allow precise control of the freezing process, thus avoiding uncontrolled freezing of soft parts and healthy bone tissue, as well as a sudden expansion of the ice front. Aim of this animal trial was to determine whether the use of modern miniature cryoprobes can avoid the above described complications.

## Methods

A commercially available cryotherapy system (Erbokryo CS-6-System, Erbe, Tübingen, Germany) was used for cryoablation. This system consists of a casing with a control board and up to 6 vacuum-isolated flexible tubes with a cryoprobe (diameter 3.2 mm) at the end. A cryoprobe creates a cold zone 3 cm long. The Erbokryo is also fitted with computer-controlled temperature sensors (diameter 1.2 mm) witch allow 6 simultaneous measurements.

The cryoprobes were introduced via an access hole 3.6 mm in diameter drilled perpendicular to the cortical substance. Temperature was measured inside the cortical substance. Two 15-minute freezing cycles were done with the probe at full power, with a 6-minute thaw in between. Prior to starting the cryoablation, the position of the cryoprobe was checked radiologically and recorded.

24 sheep with a mean weight of 61 kg (range 39–78 kg) were placed under general anaesthesia and, using a single cryoprobe introduced through a lateral access hole, one cryoablation was done in the distal diametaphyseal transitional region of the head of the medial left tibia and the right femur of each sheep. For the tibial head, a medial access hole was drilled and the cryoprobe introduced centrally 1.5 cm below the joint and pushed to the other side of the cortical substance.

For the femoral cryoablation, the cryoprobe was introduced through a distal, posterolateral access hole in the area of the linea aspera at the diametaphyseal transition. In addition, 4 temperature sensors were introduced through access holes drilled radially at 1 cm from the bore hole. The cryoprobes were only introduced 1 cm into the bone to avoid freezing the cortical substance. Thus, the necrosis zone (which roughly corresponds to the -10°C isotherm [24]) only comprised an area of 2.4 × 2.4 in the outer cortical substance. Control holes and access holes were drilled on the contralateral sides (left femur, head of the right tibia).

**Figure 1:**
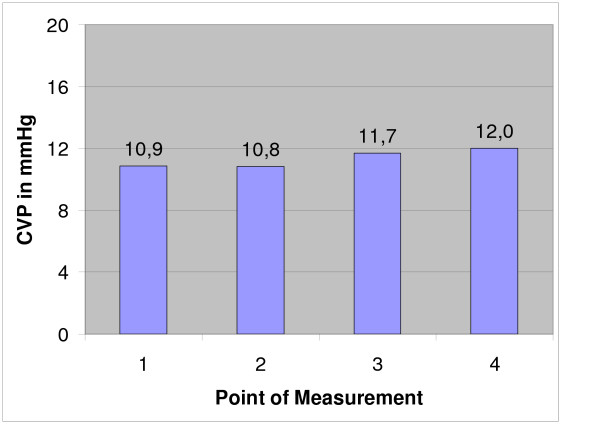
Mean central venous pressure (CVP): Measurements were done before (point 1) and after the two cryoablations (point 2, 3) and after drilling the controll holes (point 4).

**Figure 2:**
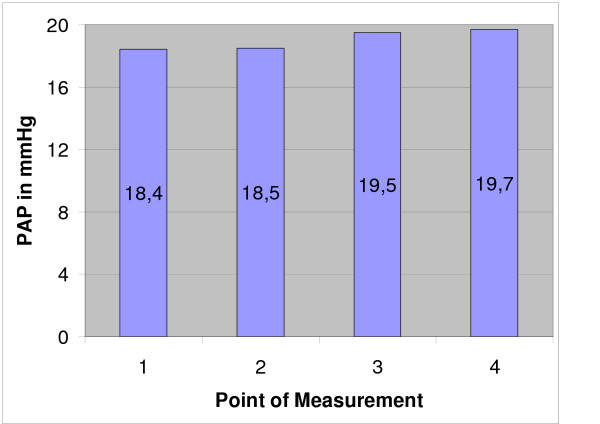
Mean pulmonary artery pressure (PAP) before (point 1) and after the two cryoablations (point 2, 3) and after drilling the controll holes (point 4).

**Figure 3:**
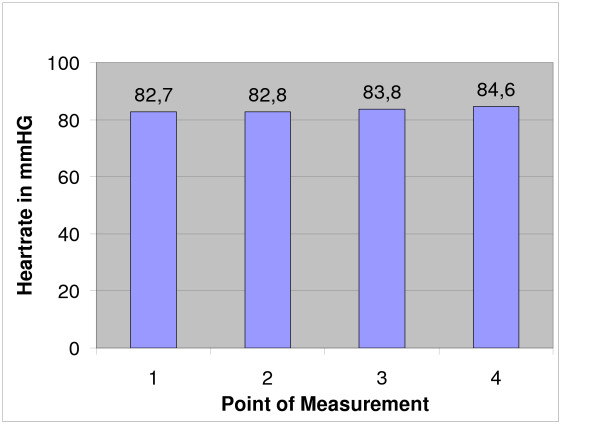
Mean heartrate measured at different times of the operation (see fig. 1, 2).

**Figure 4:**
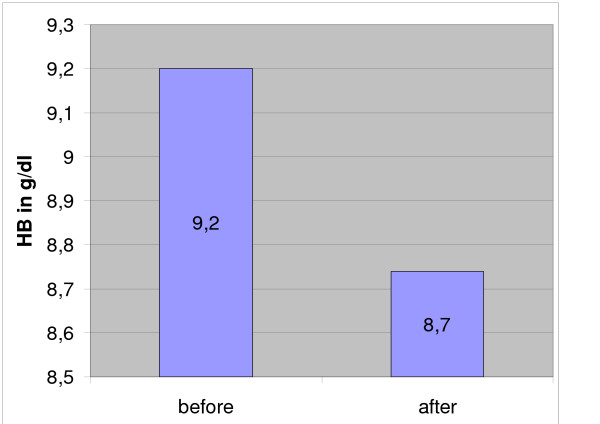
Mean hemoglobin (HB) measured before and directly after the operation.

**Figure 5:**
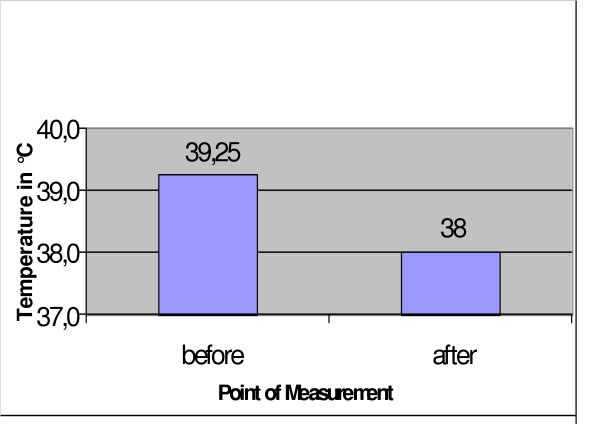
Mean deep temperature measured before and after the operation.

All operations were done under 600 mg clindamycin i.v. and 12 hours prior to surgery, each animal also received thrombosis prophylaxis (0.3 ml nadroparin calcium [Fraxiparin^®^] s.c.). There was no postop thrombosis prophylaxis since all animals were fully mobile after anaesthesia. The pulmonary-arterial pressure (PAP) and the central venous pressure (CVP) were measured via a pulmonary catheter inserted into the jugular vein. Measurements were done before the first cryoablation (1, Fig. 1, 2, 3), after the first cryoablation on the right femur (2, Fig. 1, 2, 3) and the head of the left tibia (3, Fig. 1, 2, 3), as well as after drilling the control holes immediately after shifting the animals from the right back to the left unilateral recumbent position (4, Fig. 1, 2, 3). Intra- and perioperative monitoring was complemented by measurements of heart rate (Fig. 3) and oxygen saturation via pulse oxymetry, as well as blood counts (Fig. 4), deep body temperature (Fig. 5), blood gases and electrolytes after each cryoablation. Postoperative complications were monitored clinically. 8 animals were sacrificed at 8, 16 and 32 weeks postop and tissue samples taken from the lungs and from blood vessels in the areas of cryoablation were examined for signs of embolism. Samples were taken from each lobe and from the femoral vein, fixed in formaline and stained with hematoxylin and eosin (HE). Furthermore, the ablation sites were examined histologically for infection of the soft tissue respectively osteomyelitis. X-rays were taken after the animals were sacrificed.

## Results

Mean duration of operation was 4 h 22 min, mean duration of anaesthesia 5 h 13 min. In 18 of 24 cases, the mean pulmonary-arterial pressure PAP and the mean central venous pressure (CVP) showed no significant changes at any of the 4 sampling times (Figs. 1 and 2). The PAP rose in 2 animals while in 4 other cases, the haemodynamics could not be determined for technical reasons. There was only a temporary increase of the mean PAP and CVP while the animals were in the right unilateral recumbent position, but all values normalised when the animals were shifted to their left side. The heart increased marginally during the operation (Fig. 3).

**Figure 6:**
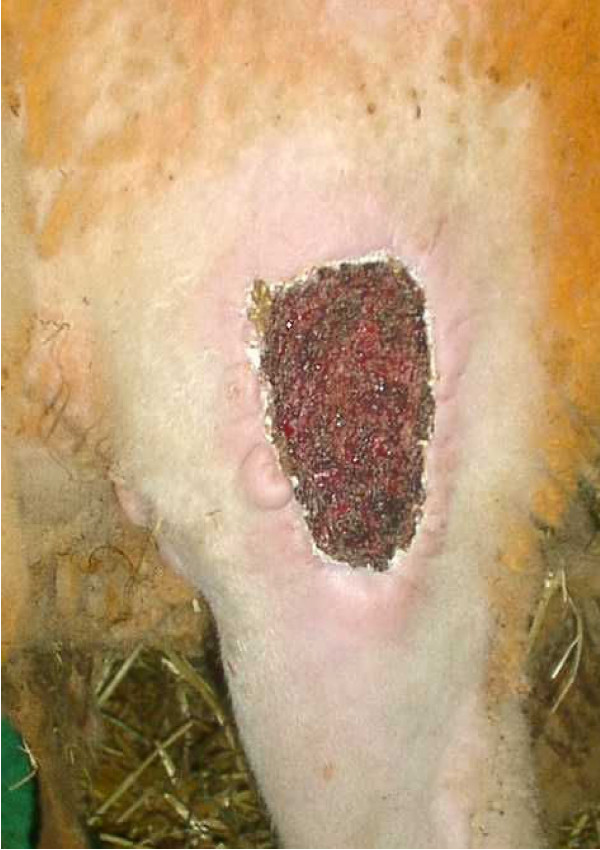
Superficial wound infection 2 weeks after cryosurgery.

Blood gas analysis showed an increase in pCO_2 _at constant oxygen saturation after the animals were shifted to their right side. In 5 of the animals, the pCO_2 _remained slightly higher even after they were shifted to their left side. PH, base excess, electrolytes and lactate showed no changes at any sampling time. Hemoglobin decreased marginally (Fig. 4). The deep body temperature (taken central-venously) before and after cryosurgery was not obviously related to any single cryoablation, but it did show a mean decrease of 1.25°C over the course of the entire operation (Fig. 5). Clinical follow-up observations yielded no remarkable findings. The mean time of recovery – i.e., from the end of anaesthesia until the animals were able to stand up again – was 86 minutes. None of the 24 animals showed any clinically significant respiratory insufficiency. One animal developed a severe wound infection which required treatment. The wound was excised and showed secondary healing under antibiotics (Fig. 6).

Autopsy revealed clean scars in all animals. There were macroscopically and microscopically no signs of ongoing or resolved osteomyelitis. All vein samples were free of old or recent thrombi. The lungs were grossly unremarkable. Histology of the bronchopulmonary arteries showed no evidence of acute or cronic embolism or thrombus. Thus, none of the animals had suffered an embolism even at the segment level. Some did show a marked bronchial pneumonia, although it followed an asymptomatic course and was therefore of no consequence. No animal showed spontaneous fractures, and X-rays taken at the end of the trial revealed no evidence of healed fractures.

## Discussion

Intravasation of adipose tissue or bone marrow can lead to acute blockage of pulmonary microcirculation, with increased resistance in the arterial pulmonary capillaries and a secondary increase of pressure in the pulmonary artery and the right atrium [22]. This causes the pCO_2 _to rise and oxygen saturation to decrease, with tachycardia resulting from left ventricular volume deficiency. To date, no comparable trials on large animals have been done examining cryoablation in bone tissue with modern cryoprobes. Kerschbaumer et al. [23] found no lung embolisms in rabbits after cryosurgery. On the other hand, Oeseburg also used rabbits and observed a large number of bone marrow embolisms in the extraosseous veins immediately after cryoablation [21].

One of the aims of this study was the detection of larger, clinically significant lung embolisms, we restricted ourselves to venously measurable hemodynamic parameters so as not to cause additional, iatrogenic complications. Likewise, we decided against a transesophageal ultrasound probe for detecting microemboli [24,25] since the animals already were administered a large transesophageal tube to aspirate gastric juices during the operation, thus leaving no room for an ultrasound probe.

None of the animals showed histological evidence of lung embolisms, nor were any embolism-specific hemodynamic phenomena or blood gas changes observed. The rise in pCO_2 _seen in all animals at the end of the operation is likely to be due to the impaired pulmonary gas exchange and decreased venous flow resulting from the increased intraabdominal pressure, which in turn can probably be attributed to the distended rumen during while the animals were lying on their right side. The rise in pCO_2 _explains the slight acidosis which the animals showed towards the end of the operation, and is therefore not to be seen as pathological.

In our opinion, the absence of significant embolisms is due to the small diameter of the probe, which prevents the intramedullar pressure from rising when the probe is introduced. A further reason could be the controlled expansion of the ice front in the bone, which prevents intramedullar pressure from peaking, and hence bone marrow or adipose tissue from being pressed out of the marrow cavity.

As expected, we did not observe any decrease in body temperature after cryosurgery as was reported for small animals such as mice and rats [26]. The minimal reduction in body temperature which we did see towards the end of the operation can be explained by the normal cooling of the body despite a heating pad. We believe that the more pronounced decrease in body temperature in small animals is due to their smaller body volume, next to which the cryoprobe is comparatively much larger. Hence, the results of our trials with large animals can be extrapolated more readily to human patients than can results from similar trials with small animals, and a significant decrease in body temperature is not to be expected in human patients.

Except for hemoglobin, all blood chemistry values remained essentially unchanged during the operation. The drop in hemoglobin by on the average 0.5 g/dl is not clinically significant.

Clinical follow-up revealed one serious wound infection, which underscores the tendency these wounds have for infection [20]. Even so, it would seem to us that the risk of infection can be controlled with perioperative administration of antibiotics, as is also evident from the histology of the treated bone sections, none of which developed acute inflammation. Nevertheless, it must be admitted that the tissue treated here was healthy bone in animals with an intact immune system, and a higher infection rate must be expected when applying this method to patients with advanced malignancy.

Convalescence after cryosurgery is associated with changes in bone stability, a topic which few studies have addressed so far. Gage et al. (1967) reported 11 spontaneous fractures in 20 dogs, where the entire cross-section of the femur was frozen over a length of 4.5–7 cm [9]. Further studies report a maximum reduction in bone stability some 8 weeks after cryosurgery [27,20]. The absence of fractures in our trial shows that limiting bone necrosis by controlled freezing and minimum tissue loss when introducing the cryoprobe helps minimise the reduction in bone stability, and hence prevent fractures. To be sure, the size of a tumour dictates the extent of the freezing zone, so that stabilising measures may be necessary.

## Conclusion

In conclusion the use of modern miniature cryoprobes for cryoablation of bone tissue seems to be a gentle method, and the complications reported for earlier systems did not occur in this study. Therefore, this cryosurgical technique has a pontential in human subjects and could be usefull either to complement conventional resections, or else as a minimally invasive alternative procedure.

## Competing interests

The author(s) declare that they have no competing interests.

## Authors' contributions

FP and PM carried out the operations and drafted the manuscript. MB and TK carried out the hole perioperativ treatment of the animals and the monitoring throughout the intra- and perioperative phase. HE performed the histological investigations. JHF participated in the design of the study and performed the statistical analysis. PE conceived of the study, and participated in its design and coordination. All authors read and approved the final manuscript.

## Pre-publication history

The pre-publication history for this paper can be accessed here:


